# A Case of Stage I Vulvar Squamous Cell Carcinoma with Early Relapse and Rapid Disease Progression

**DOI:** 10.1155/2019/1018492

**Published:** 2019-07-03

**Authors:** Marta Peri, Antonino Grassadonia, Laura Iezzi, Patrizia Vici, Michele De Tursi, Clara Natoli, Nicola Tinari, Marinella Zilli

**Affiliations:** ^1^Medical Oncology Unit, Department of Medical, Oral and Biotechnological Sciences, University “G. D'Annunzio”, Chieti, Italy; ^2^Division of Medical Oncology 2, IRCCS Regina Elena National Cancer Institute, Rome, Italy; ^3^Medical Oncology Unit, SS Annunziata Hospital, Chieti, Italy

## Abstract

Squamous cell carcinoma (SCC) is the most common subtype of vulvar cancer. Locoregional surgery is often curative when the tumor is diagnosed at an early stage. However, the disease can unexpectedly evolve with a dismal prognosis even after an early diagnosis. We report a case of a woman who experienced a rapid, chemorefractory tumor progression after surgery for stage IB vulvar SCC.

## 1. Introduction

Vulvar cancer is the fourth most common gynecologic cancer after endometrial, ovarian, and cervical cancer, accounting for about 5% of all female genital tract malignancies [1].

The most common histological type of vulvar cancer is squamous cell carcinoma (SCC), which accounts for about 90% of the cases. Two different types of SCC have been described: a keratinizing form and a warty/basaloid form [2]. The former occurs predominantly in postmenopausal women with a background of lichen sclerosus or lichen planus evolving in differentiated vulvar intraepithelial neoplasia (d-VIN) and is associated with poorer prognosis [3, 4]. The latter is more common in younger patients and is related to high-risk papillomavirus (HPV) infection evolving in high-grade squamous intraepithelial lesion (HSIL or VIN3) [3, 5].

Radical local excision with inguinofemoral lymph node dissection currently represents the standard of treatment for women with vulvar cancer [6]. Since surgery is associated with high morbidity, noninvasive methods are commonly utilized to evaluate the extension of disease in the preoperative setting. In particular, magnetic resonance imaging (MRI) is helpful for the assessment of local tumor extension and inguinal lymph node involvement [7].

The dissemination pattern of vulvar carcinoma is mostly lymphogenic, and the inguinofemoral lymph nodes are the primary site of regional spread [8]. Distant spread usually occurs late in the course of the disease. In the absence of distant metastases, the most important prognostic factor is represented by pathologic status of the inguinal nodes, while the size of the primary tumor is less important in defining prognosis [9].

Herein, we describe a case of vulvar carcinoma diagnosed at stage IB FIGO that displayed a very aggressive behavior. The patient experienced early local recurrence and rapid metastatic disease progression causing death just a few months after relapse.

## 2. Case Presentation

A 70-year-old woman presented at the gynecology unit of our hospital complaining about a painful vulvar lesion in May 2017. She had no significant medical history. Physical examination revealed an exophytic and ulcerative vulvar mass, approximately 4 cm in diameter, localized on the right labium majus at less than 2 cm from the midline, without palpable inguinal lymph nodes bilaterally. An incisional biopsy was performed, and histology revealed an invasive poorly differentiated vulvar SCC. A total-body CT scan performed to stage the disease resulted negative for distant metastases.

The patient underwent right hemivulvectomy in order to obtain wide tumor-free pathological margins in June 2017. Concomitant inguinal lymph node dissection was not performed due to the patient's refusal (risk of lymphedema). Histopathologic findings confirmed a poorly differentiated vulvar SCC arising on a background of lichen sclerosus. The size of the invasive SCC lesion was 4.5 cm with a depth of invasion of 2.7 mm and no lymphovascular invasion. All surgical margins of the lesion were tumor-free (more than 1 cm).

She was addressed to our oncology unit in July 2017. We required a disease restaging by abdominal and pelvic MRI scan and chest CT scan. No evidence of distant metastases resulted from the imaging studies. Therefore, we suggested locoregional lymph node dissection in order to define the pathologic stage of the tumor and to plan postoperative adjuvant radiotherapy to the groin just in case of lymph node involvement.

In August 2017, a bilateral inguinofemoral lymph node dissection was performed with all nodes (twelve) resulting negative for metastatic spread on conventional hematoxylin-eosin staining. The tumor was staged as FIGO stage IB, and the patient was addressed to strict follow-up.

However, just one month later (September 2017), the patient developed a local recurrence with a 3 cm nodule in the right vulvar area and a 0.8 cm lesion in the clitoris. A wide local excision was performed and histopathology examination revealed a poorly differentiated vulvar SCC in both lesions.

A restaging CT scan of the chest, abdomen, and pelvis showed multiple bilateral pulmonary metastases and multiple inguinal and pelvic lymph node involvement.

**Figure 1:**
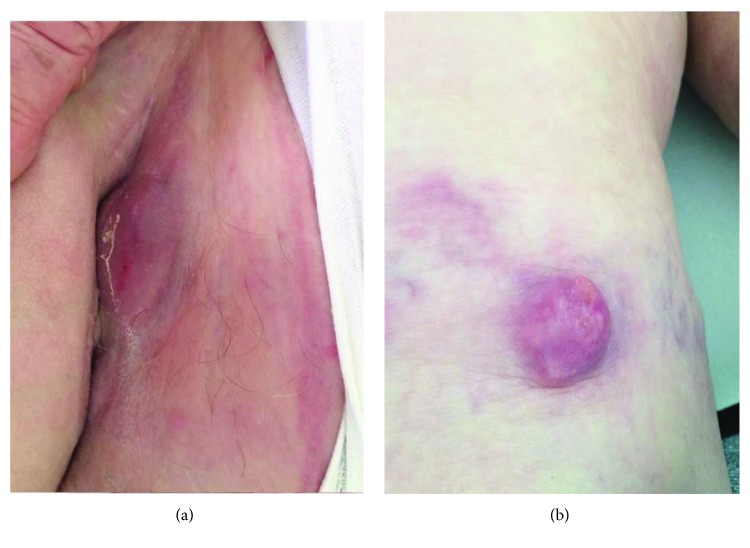
Cutaneous tumor progression during the first-line chemotherapy. Erythematosus nodules on the skin of the right groin (a) and thigh (b).

Because of recurrence, systemic chemotherapy was started with carboplatin (AUC5 day 1 every 3 weeks) and paclitaxel (80 mg/m^2^ days 1 and 8 every 3 weeks). After 3 cycles, a total body CT scan showed progression of metastatic disease in the lungs, lymph nodes, and liver. Moreover, a painful erythematosus nodule appeared on the skin of the right groin (Figure 1(a)) and right thigh (Figure 1(b)).

**Figure 2:**
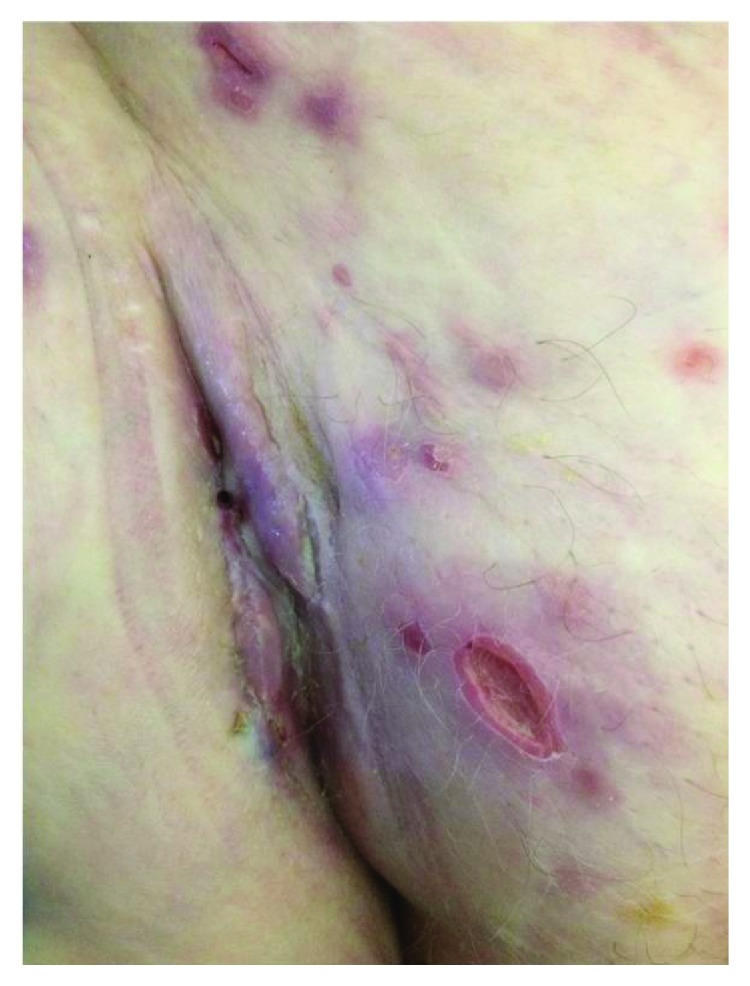
Further cutaneous progression during the second-line chemotherapy. Ulceration and fistulization of the right groin nodule.

Because of disease progression, a second-line chemotherapy with capecitabine (1000 mg/m^2^ bid, days 1-14 every 21 days) was started (December 2017). After 3 cycles of treatment, the patient presented ulceration and fistulization of the groin lesion (Figure 2) and new skin nodules in the right thigh associated with extremities lymphedema. She complained of perineal pain and analgesic therapy was prescribed. Moreover, palliative radiotherapy to inguinal metastases (30 Gy in 10 fractions) was performed.

**Figure 3:**
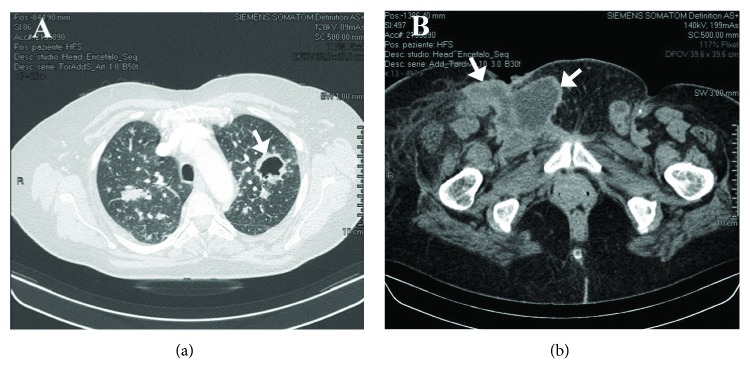
Further systemic tumor progression during the second-line chemotherapy. CT scan showing multiple lesions in the lungs (a), some excavated (arrow), and matted metastatic lymph nodes in the iliac/inguinal region (b) (arrows).

A reevaluation CT scan (February 2018) revealed further progression of the disease with multiple liver metastases, multiple excavated lesions in the lungs (Figure 3(a)), and matted metastatic iliac/inguinal lymph nodes (Figure 3(b)).

The patient died one month later, in March 2018, because of respiratory failure.

## 3. Discussion

We described a case of vulvar SCC that, despite an early-stage presentation at diagnosis, rapidly evolved into metastatic chemoresistant disease, leading the patient to death in a few months.

Vulvar SCC is a rare disease mainly occurring in postmenopausal women. The prognosis for patients with early-stage disease is generally good, but cancer can spread from its original site to locoregional nodes and/or distant organs by lymphatic embolization or hematogenous diffusion, respectively. Lymphatic spread represents an early event in the course of the disease and involves ipsilateral inguinal, femoral, and pelvic lymph nodes, usually in a sequential manner. Lymph node status remains the single most important prognostic factor. The 5-year overall survival is more than 90% for patients without nodal involvement, which reduces to less than 60% in the case of nodal involvement [9].

Our patient presented with a negative pathological nodal status at diagnosis, but unfortunately, her disease-free survival was very short, with local relapse occurring just one month after surgery. It is evident that other prognostic factors other than stage may influence the natural history of the disease. For example, tumor characteristics such as high-grade or lichen sclerosus-related etiopathogenesis, as in the case herein described, could play a pivotal role in determining a poor prognosis [4, 10].

Hematogenous spread to distant sites, including the lung, liver, and bone, usually occurs late in the natural history of the disease. Cutaneous metastases from vulvar carcinoma have been rarely described, but when documented, they are associated with short survival [11–15]. The median time to death from the diagnosis of skin metastasis has been estimated to be around 6 months [4, 15].

In our case, distant metastases developed shortly after diagnosis, in an early infiltrating phase of the disease (only 2.7 mm). Skin metastases also manifested early during the metastatic phase, in the course of the first-line systemic treatment. Consistent with literature data, the appearance of cutaneous metastases was a predictor of short survival for our patient. She died after only 4 months.

The rapid tumor progression was accompanied by a chemoresistant phenotype with a dismal prognosis. In fact, disease progressed during the first-line platinum-based chemotherapy in all the secondary sites and further progressed during the subsequent second-line treatment with capecitabine.

To date, the therapeutic management of metastatic vulvar SCC is heterogeneous and data are insufficient to recommend a preferred chemotherapeutic regimen in the palliative setting. Usually, regimens with proved efficacy in advanced cervical or anal cancers are chosen for the treatment of metastatic vulvar SCC [6].

Advances in the molecular biology of vulvar SCC may provide insight into the future management of this tumor [16]. At the moment, despite the two distinct etiologies, there is no specific recommended chemotherapeutic regimen for HPV-associated or HPV-independent tumors.

## 4. Conclusion

Vulvar SCC can unexpectedly evolve with a dismal prognosis even if diagnosed at an early stage. The disease can be rapidly progressive and refractory to chemotherapy. Novel therapeutic approaches are required.
